# Youth-to-senior transition in women’s and girls’ football: Towards a better understanding of relative age effects and gender-specific considerations

**DOI:** 10.1371/journal.pone.0283781

**Published:** 2023-05-04

**Authors:** Paolo Riccardo Brustio, Roberto Modena, Gennaro Boccia, Matteo Vogliazzo, Adam Leigh Kelly

**Affiliations:** 1 Department of Clinical and Biological Sciences, University of Turin, Turin, Italy; 2 NeuroMuscularFunction Research Group, School of Exercise & Sport Sciences, SUISM University, Turin, Italy; 3 Faculty of Health Sciences and Social Care, Molde University College, Molde, Norway; 4 CeRiSM (Sport Mountain and Health Research Centre), University of Verona, Verona, Italy; 5 School of Exercise & Sport Sciences (SUISM), University of Turin, Turin, Italy; 6 Centre for Life and Sport Sciences (CLaSS), Faculty of Health, Education, and Life Sciences, Birmingham City University, Birmingham, West Midlands, United Kingdom; Instituto Politécnico de Santarém: Instituto Politecnico de Santarem, PORTUGAL

## Abstract

This study aimed to evaluate youth-to-senior transition and the relative age effect in Italian female football national teams. Birthdate data of 774 female players selected for Under 17 (N = 416), 19 (N = 265) and National Senior team (N = 93) were analysed. The youth-to-senior transition rate was determined by the number of youth players competing in the Senior National team (and vice versa), whilst birth quarter (Q) distributions with a chi-square goodness-of-fit test. Only 17.4% of youth players were able to be selected for the Senior National team, whereas 31.2% of the players reached the high-senior level without being selected for youth age groups. Data revealed a skewed birth date distribution in Under 17 and 19 teams (on average, Q1 = 35.6% vs Q4 = 18.5%) but not in the Senior National team. Youth players born in Q1 were two times more likely to be selected than in Q4. In Under 17, goalkeepers, defenders, and midfielders of Q1 players were overrepresented. However, Q4 players recorded higher conversion rates than Q1 (Q1 = 16.4% vs. Q4 = 25.0%). National youth experience may not be a prerequisite for being selected at the senior level. Moreover, this confers a higher probability of playing in the National Senior team than players not selected in youth rosters.

## Introduction

Football represents the most popular sport worldwide, with female participation significantly increasing over the last two decades [1]. This has coincided with the Federation Internationale de Football Associations (FIFA) who have invested finances and resources to promote football participation in girls and women [2]. Although the number of studies involving female players is also slowly rising [3], it remains considerably lower than those focused on males [4]. Talent identification and selection is not an exception [5], as a recent review [6] showed that among 27 studies, few involved both male and female players, and only two involved female-only football players [7, 8]. Of these female-only studies, the prognostic validity of physical during adolescence (i.e., 20 m sprint or dribbling ability) to predict youth [8] or senior success [7] was shown in German female football. Nevertheless, the application of early predictors is often flawed and subject to biases, which limits academies’ success in identifying correctly future successful and talented players [9–14].

Studies on male players have showed that practice experience gained from youth international teams is a limited predictor of senior success [12, 13]. Indeed, professional academies show a high turnover of youth players [15], mainly caused by repeated procedures of selection and de-selection throughout childhood, adolescence, and early adulthood [9]. In this regard, a study on the male Italian football talent system identified as, independent from playing position, 10–20% of players selected youth national teams (i.e., Under 16, 17, 18, or 19) were subsequently selected in senior national team [16]. This suggests that international youth experience may not be a prerequisite for an international career in adulthood [14, 16].

One of the most prominent and influential factors that may create biases in talent selection and identification in football is the relative age effect (RAE) [5, 6, 17]. The RAE reflects the (dis)advantage created by the interaction between the chronological age and the selection cut-off dates (e.g., January to December). Given youth football categories are banded according to the annual or biennial-age groups, players within the same age group are up to twenty-four months apart in chronological age. Consequently, relatively older players (i.e., players born in the first quartile of the years; Q1) may benefit from anthropometric, physiological, and psychosocial advantages that facilitate superior sports performance, and, as such, are considered more gifted and talented than relatively younger players (i.e., players born in the fourth quartile of the years; Q4). Therefore, supported by a traditional subjective assessment, coaches and talent scouts may consciously or unconsciously select relatively older rather than relatively younger players. This is true, particularly in youth football, where the invasive nature combined with the high physical demand required during competition may exacerbate the RAE [18, 19] and distort the identification, selection, and successful transition of players [20].

While the RAE is well documented in male football, whereby a consistent and pervasive RAE exists, especially in the youth age groups [14, 18, 19], its presence remains inconclusive in females [17, 21]. More specifically, the RAE seems mixed depending on contextual factors such as sociocultural context (i.e., depth of competition, attraction level and country-specific differences) [22], age groups [20], competition levels [23], playing positions [20], and historical moment [24]. For example, there was a significant difference in quartile distributions across elite German national teams [25] (i.e., Q1~31% vs. Q4~21%). However, when RAEs were investigated based on competition level, it was only prevalent in second tiers (i.e., second league) and not within high-level senior (i.e., first league) or youth cohorts (national selection). Similarly, high-level senior French players did not present the RAE [26] whereas players born near the selection date in Italy were about 1.62 times more likely to reach the high-level tiers [27]. Likewise, when considering Women’s Football World Cup rosters (years between 2008–2019), the RAE effect sizes have been identified only in the U17 and U20 tournaments but not in senior tournaments [20, 24]. In particular, for the Under 17 and 20 age groups, a RAE was observed (i.e., Q1~33% vs. Q4~20%), especially when considering midfielders (i.e., Q1~37% vs Q4~17%) [20]. Differently, Andrew et al. [21] found no RAE in UEFA European tournaments of U17, U19, and senior age groups between 2019–2022, whilst Barreira et al. [28] reported no RAE in Olympic tournaments between 1996–2016. On the other hand, the few analyses on the national academy talent system (i.e., Swiss and U.S.) showed that the RAE is dependent by competition levels [29, 30]. For example, while the proportion of relatively older players was higher in the talent development programme (i.e., Q1~28% vs. Q4~18%), the proportion of birth distribution between Q1 and Q4 was similar in the national level selection (i.e., OR = 1.55 [0.83, 2.90]). Hence, it can be stated that the RAE magnitude in females is sparse, reveals contradictory results, and may be affected by geographical zones (i.e., sociocultural contexts) [23].

Although the RAE impacts the selection of future football players, with the potential cost of missing this talent may be difficult to calculate accurately (Kelly et al., 2020), some studies provide evidence of a possible long-term advantage of relatively younger players during their senior career. This scenario, referred to as the ‘underdog hypothesis’ [31, 32], suggests that players born towards the end of the selection date have a greater opportunity to successfully transition to senior level once selected into talent pathways [14, 33]. In this regard, recent studies showed that the probability of achieving a professional contract was about four times higher for English relatively younger players [33], as well as about three and four times the probability of competing in the Italian Senior National Team or a UEFA European Champions and/or FIFA World Championship respectively [14]. Interestingly, in the Italian context, the RAE magnitude was smaller for players that successfully transitioned from youth to senior national teams than those who failed to transition [16]. On the other hand, being a relatively younger player should essentially facilitate long-term development and confer a significant potential for success at the adult level, including the enhancement of skill proficiency (e.g., superior technical and tactical skills) and superior psychological and social skills necessary to overcome the odds of the RAE [31–34].

Summing up, the study of successful youth-to-senior transition in female football players remains unclear and is yet to be investigated. Moreover, the RAE research often focuses on top European clubs or international competitions, whilst the national system is less studied, which should also be considered to the extent to which the RAE is rooted. Additionally, all reports on female football have investigated the phenomenon at a one-time point without considering the players’ career trajectories, leading to limited knowledge about the relationship between birthdates and the likelihood of successful transition from the youth-to-senior levels. Thus, to extend the our knowledge regarding successful youth-to-senior transition and RAE, whilst also considering the possible influence of the underdog hypothesis in female football players, the purposes of the present study were (*Part I*) to evaluate successful and unsuccessful transition from youth-to-senior level, (*Part II*) to comprehensively quantify the prevalence and magnitude of RAE, considering also playing position, and (*Part III*) to evaluate quartile rate on this transition in Italian football national teams.

## Materials and methods

Data regarding Italian female players was downloaded through the open-access online databases provided by the Federazione Italiana Giuoco Calcio (FIGC, https://www.figc.it) in November 2022. This source provides players selected at least once for the Youth National Teams (i.e., Under 16, Under 17, Under 19, and Under 23) and the Senior National Team. The FIGC only introduced the Under 16 and 23 age groups in 2015. For this reason it was difficult to rebuild the whole carrer profiles of these players and thus, to avoid possible bias expecially when considering the youth-to-senior transition rate, these two age groups were not considered for this study. Data extraction included each player’s name, birthdate, and playing position. When the database did not provide the birthdate, manual searching of athletes through other sources (i.e., https://www.transfermarkt.it/) was performed. We considered all players born from 1985 to 2006 (both years included). Overall, 774 players (Under 17 = 53.7%; Under 19 = 34.2%; Senior = 12.0%) were included in the final database for analysis of RAEs (of note, an athlete could be present in more than one age group depending on how many times she was selected). For the analysis of the youth-to-senior transition rate (Part I and Part III), after removing duplicated players, we considered a subsample (N = 268) including only players born from 1985 to 1998 (both years included). Therefore, only players with the whole career presented were considered. Due to the study data being in the public domain, no informed consent and approval by an Ethical Committee were required.

### Analysis

*Part I*: prospectively, we calculated the proportion of players selected in the Under 17 or 19 age group or both and then selected in the National Senior team. Retrospectively, we calculated the proportion of players selected in the National Senior team and also selected the Under 17 or 19 age group or both. The transition rates were calculated using a binomial proportion confidence interval [90% CI].

*Part II*: for each player, birth quartiles were calculated according to the cut-off selection date of the Italian Federation (i.e., 1^st^ January). Thus, Quartile 1 (Q1) identified players born between January and March, Quartile 2 (Q2) players born between April and June, Quartile 3 (Q3) players born between July and September, and Quartile 4 (Q4) players born between October and December. For each age cohort, observed quartile distributions were compared to expected quartile distributions using Chi-Square Goodness of Fit tests (χ^2^) with a p-value set at 0.05. Expected quartile distributions were calculated from the average national live birth. Effect size magnitudes were determined by Cramer’s V and interpreted as follows: 0.06 ≤ V trivial, 0.06 < V ≤ 0.17 small, 0.17 < V < 0.29 medium, and V ≥ 0.29 large effect. Odds ratios (ORs) and 95% confidence intervals (CIs) were calculated to compare the first and the fourth quartile (i.e., Q1 vs Q4) and between the first and second semester (i.e., S1 vs S4, half-year distribution comparisons). The above analyses were performed separately for each age group, considering playing positions together and separately for goalkeepers, defenders, midfielders, and forwards.

*Part III*: Similar to the approach used in *Part I*, prospectively and considering only successful youth players (i.e., those who subsequently made a National Senior team appearance), we quantified the youth-to-senior transition rates among the different birth quartiles. The transition rates were calculated for each birth quartile using a binomial proportion confidence interval [90% CI]. Moreover, observed quartile transition rate distributions were compared to expected quartile distributions (i.e., quartile distribution calculated based on youth players) using Chi-Square Goodness of Fit tests (χ^2^), with Cramer’s V magnitudes and ORs calculated.

All the analysis was performed using a custom script written in MATLAB R2020b (MathWorks, Natick, Massachusetts), while the Sankey diagram was prepared by an online tool (https://sankeymatic.com/build/).

## Results

### *Part I*. Youth-to-senior transition rate

#### Prospective analysis

**Fig 1:**
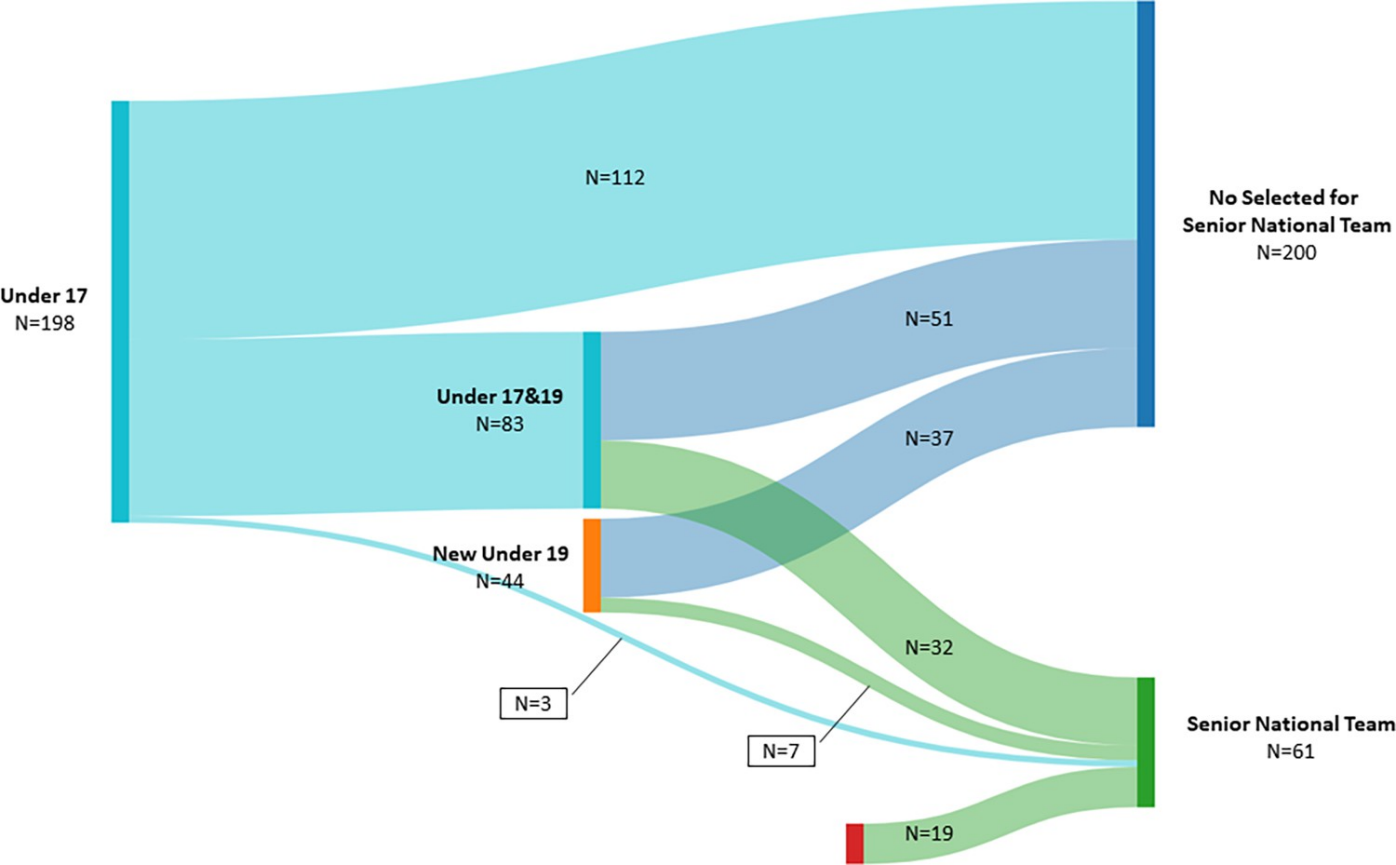
Youth-to-senior transition rate. Overall visual inspection of the youth-to-senior transition rate. The Sankey diagram provides the number of players able to reach the National Senior team from Under 17, 19 (or both together). The figure also provides the number of players that were not selected for Senior National Team.

The career pathway of 261 players was considered for the youth-to-senior transition rate. Of this sample, 242 players were selected in the youth age groups (i.e., Under 17 and 19), while only 19 players were selected in the National Senior team. Only 42 players (17.4% [12.8, 22.7]) selected in the youth reached the senior level. Considering the two age groups evaluated, the transition rate was 17.7% [13.4, 22.7] and 30.7% [24.0, 38.1] for those Under 17 and 19, respectively. A total of 38 players (41.9% [36.0, 48.0]) transited from Under 17 to 19, and of these, 32 players (16.2% [12.0, 21.1]) reached the National Senior team. On the other hand, only three (i.e., 1.5% [0.4, 3.9]) and seven players (i.e., 5.5% [2.6, 10.1]) were able to directly reach the National Senior team from the Under 17 and 19 age group, respectively. Fig 1 offers an overall visual inspection of the overall youth-to-senior transition rate.

#### Retrospective analysis

Out of 61 players that were able to reach the National Senior team, nineteen players (31.2% [21.5, 42.3]) were not selected from the youth national selection, while 42 players (68.9% [55.7, 80.1]) were selected from the youth national selection. Specifically, 52.5% [41.2, 63.5] of the players were selected both Under 17 and 19, 4.9% [1.4, 12.2] only Under 17, and 11.5% [5.5, 20.5] only Under 19 age groups.

### *Part II*. Relative age effect

Table 1 reports the birth quartile distribution, Chi-Square (χ^2^) statistics, and the ORs for all players selected in the Under 17, Under 19, and National Senior team (i.e., playing positions together) as well as considering playing positions (i.e., goalkeepers, defenders, midfielders, and forwards).

**Table 1:** Birth quartile distribution, chi-square value, and odds ratio analysis considering the different playing positions.

Age group	Playing Position	Total N	Q1%	Q2%	Q3%	Q4%	*χ* ^*2*^	P	*V*	*ES cat*.	Q1vsQ4	S1vsS2
**Under 17**	All Players	416	36.3	25.0	22.1	16.6	41.789	**<0.001**	0.18	Medium	2.28 [1.53, 3.38]	1.58 [1.20, 2.09]
	Goalkeepers	45	35.6	22.2	28.9	13.3	4.720	0.194	0.19	Medium	2.77 [1.04, 7.38]	1.37 [0.73, 2.55]
	Defenders	130	37.7	33.8	14.6	13.8	28.391	**<0.001**	0.27	Medium	2.83 [1.54, 5.19]	2.51 [1.64, 4.07]
	Midfielders	134	38.8	20.9	24.6	15.7	17.871	**0.001**	0.21	Medium	2.58 [1.45, 4.59]	1.48 [1.00, 2.28]
	Forwards	98	32.7	21.4	25.5	20.4	4.602	0.203	0.13	Small	1.66 [0.89, 3.10]	1.18 [0.76, 1.91]
**Under 19**	All Players	265	32.8	24.2	22.6	20.4	13.090	**0.004**	0.13	Small	1.69 [1.04, 2.74]	1.32 [0.94, 1.87]
	Goalkeepers	32	31.3	28.1	18.8	21.9	1.750	0.626	0.14	Small	1.50 [0.54, 4.17]	1.46 [0.69, 3.08]
	Defenders	82	32.9	24.4	22.0	20.7	4.546	0.208	0.14	Small	1.66 [0.83, 3.34]	1.34 [0.81, 2.31]
	Midfielders	82	34.1	22.0	24.4	19.5	5.445	0.142	0.15	Small	1.83 [0.91, 3.71]	1.28 [0.78, 2.18]
	Forwards	68	32.4	25.0	23.5	19.1	3.413	0.332	0.13	Small	1.77 [0.82, 3.82]	1.34 [0.78, 2.38]
**Senior**	All Players	93	25.8	31.2	17.2	25.8	5.031	0.170	0.13	Small	1.05 [0.46, 2.36]	1.33 [0.74, 2.37]
	Goalkeepers	12	16.7	41.7	8.3	33.3	0.333	0.564	0.10	Small	0.52 [0.09, 3.15]	1.40 [0.41, 4.74]
	Defenders	27	33.3	29.6	11.1	25.9	3.929	0.269	0.22	Medium	1.34 [0.43, 4.24]	1.70 [0.70, 4.50]
	Midfielders	30	23.3	33.3	20.0	23.3	1.786	0.618	0.14	Small	1.05 [0.31, 3.47]	1.31 [0.57, 3.16]
	Forwards	24	25.0	25.0	25.0	25.0	0.0	1.000	0.0	Trivial	1.05 [0.29, 3.74]	1.00 [0.41, 2.55]

Notes: Q1, first quartile percentage; Q2, second quartile percentage; Q3, third quartile percentage; Q4, fourth quartile percentage; χ2, chi-square value; P, p valueV, Cramer’s V effect size; OR, odds ratio and 95% confidence intervals [95% CI]; Q1 vs. Q4, first versus the last quartile; S1 vs. S2, first versus the last semester.

When considering all playing positions together, a birth-skewed distribution was observed both in the Under 17 (χ^2^ = 41.789, p < 0.001) and Under 19 (χ^2^ = 13.090, p = 0.004) age group with corresponding medium to small effect size (effect size ranged = 0.13, 0.18). Accordingly, the likelihood of being selected in Q1 was higher than in Q4 (OR = 2.28 [1.53, 3.38] and 1.69 [1.04, 2.74] in Under 17 and Under 19, respectively.

For the National Senior team, analysis detected an even quartile distribution compared to the expected distribution (χ^2^ = 5.031, p = 0.170). No significant odds ratio was apparent (OR = 1.05 [0.46, 2.36]).

When analysing distributions according to playing positions, for Under 17, the RAE was evident in defenders and midfielders but not in goalkeepers and forwards. The peak RAE was in the defenders, where 71.5% were born in the year’s first half (OR = 2.51 [1.64, 4.07]). No RAE was observed in the Under 19 and National Senior teams. However, overall, the trend showed that the players born in the year’s first half were overrepresented compared to the second ones (overall mean = 28.6% vs 21.3%). See the supplementary file for a visual inspection of the overall players’ data according to playing positions (i.e., S1 Fig).

### *Part III*. Youth-to-senior transition rate and birth quartile

**Fig 2:**
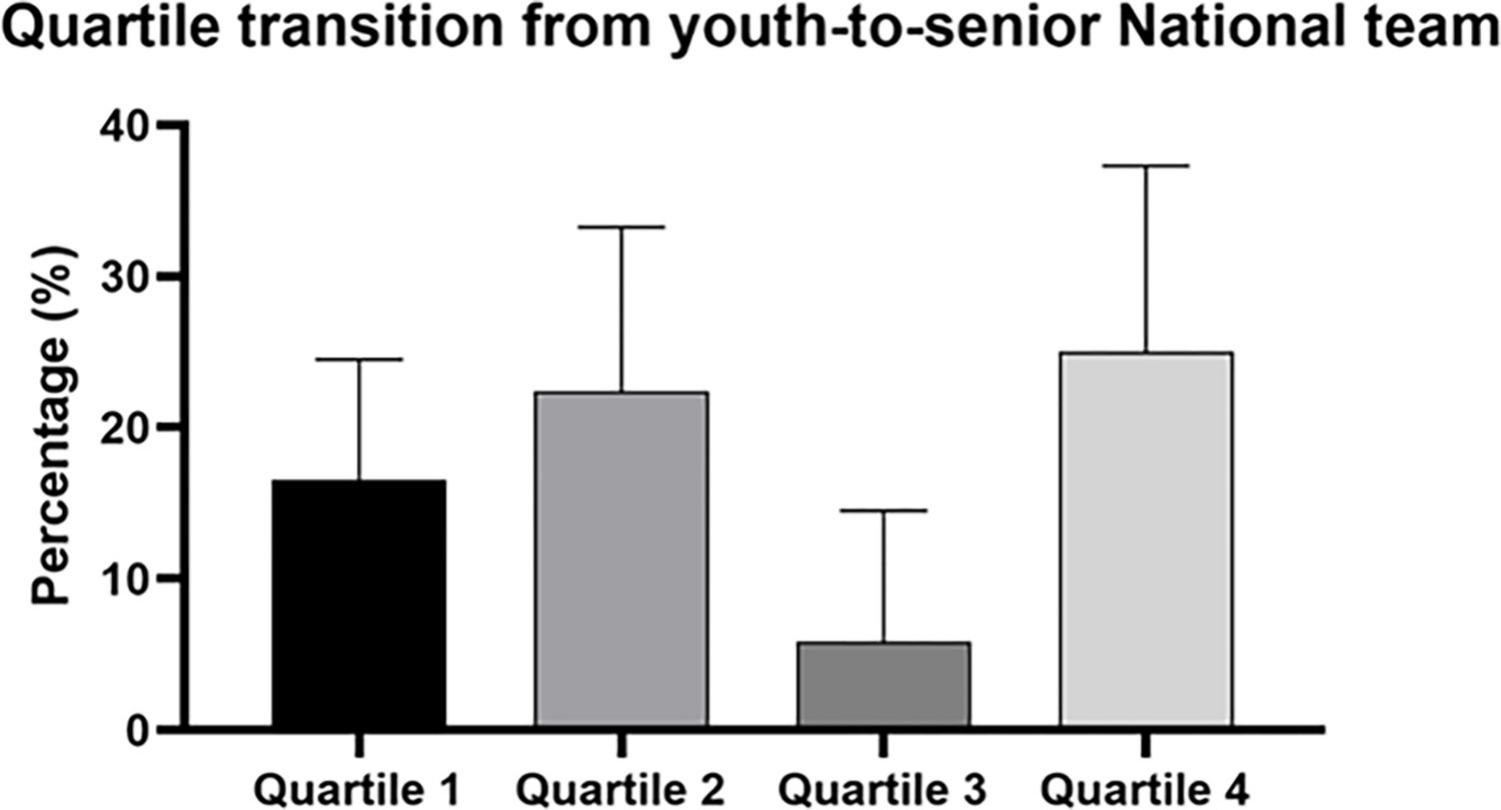
Birth quartile youth-to-senior transition rates. Data are presented calculated considering all players that competed in Under 17 or 19 and successfully reached the National Senior team.

Fig 2 shows the youth-to-senior transition rate according to birth quartiles.

Relatively younger players recorded higher conversion rates than relatively older players. Specifically, in the transition from youth-to-senior level, a larger proportion of players born in Q4 successfully transitioned out of the youth national teams to play for the senior national team (Q1 = 16.4% [10.2, 24.5] vs Q4 = 25% [15.1, 37.3]). Again, Q2 showed a similar transition rate to Q4 (i.e., 22.4% [13.8 33.3]), while Q3 showed the lowest (i.e., 5.8% [1.6, 14.5]). The successful quartile distributions were significantly skewed compared to the U17 distribution with a medium effect size (χ^2^ = 8.00, p < 0.07, V = 0.25). However, no significant odds ratio was apparent in the Q1 vs Q4 comparison (OR = 0.62 [0.19, 1.74]). The only significant OR was found between Q3 and Q4 players, with players in Q4 more likely to attain senior status (OR: 5.14 [1.03, 25.60]).

## Discussion

This study aimed to explore the successful transition from youth-to-senior level (*Part I*), investigate the prevalence and magnitude of the RAE in Italian national teams (*Part II*), and verify if quartile distribution affect this transition (*Part III*). The key findings of the study were that: (a) overall, only about 17% of youth players were able to be selected at the high-senior level, whereas only about 31% of the players reached the high-senior level without being selected at youth age groups (*Part I*), (b) data revealed a skewed birthdate distribution favouring relatively older players in Under 17 and 19 (on average, Q1 = 35.6% vs. Q4 = 18.5%), although not in National Senior team (*Part II*), and (c) the youth-to-senior transition rate is partially modulated by birthdate with the higher transition of players in Q4 according to underdog hypothesis (*Part III*).

Prospective analysis of the youth-to-senior transition rate suggests that youth national team selection might not necessarily translate into a successful transition into the senior Italian national team [16]. Specifically, approximately 18% of players selected in both youth age-group reached the senior level. Nevertheless, it is necessary to highlight that the success rate was higher as age increased (i.e., about 18% and 31% when considering Under 17 and 19, respectively). Interestingly, this data was relatively higher if compared with data of the Italian male national team, where the overall youth-to-senior transition rate of about 15% [16], which corroborates the idea that a relatively higher proportion of successful female athletes could maintain the same level during their senior career [35, 36].

The rate of players that could transition from only one youth category to the National Senior team was relatively lower (i.e., about 2 and 6% for those Under 17 and 19, respectively), suggesting that to be selected both in Under 17 and 19 may confer more chance to be selected in the national senior team. Conversely, the retrospective analysis underlined that two-thirds of the senior players were not selected from the youth national selection. This rate was higher than in the male Italian talent system, where about 20% and 40% of players Under 19 and 21, respectively, reached the national senior teams [16]. On the other hand, data underlined that in the female context, probably due to the lower depth of competitions and competitors, higher level youth experience may confer more probability to be selected at the senior level. Considering both prospective and retrospective analysis together, the data suggested that successful youth players have a low probability of being selected for the national senior team. Still, this probability is higher compared to players that were not selected during youth. Overall, the data suggested that being selected for youth teams is a prerequisite, but it is insufficient to compete in the national senior team.

Consistent significant overall asymmetries in relative age within Under 17 and 19 were observed with small/medium effect size. Findings revealed a skewed birthdate distribution favouring relatively older players (i.e., approximately 35% and 18% in Q1 and Q4, respectively, when merging Under 17 and 19). Players born in Q1 were 2.3 and 1.7 times more likely to be selected than Q4 in Under 17 and 19. According to the current literature, the RAE magnitude decreased as age increased [17, 18]. Overall results indicated that, like male Italian football players, relatively older female players are more likely to enter national youth selection during the youth academic pathway [14]. Again, this data may probably be explained because players’ selection is based on the current level of performance rather than on long-term performance. Nevertheless, these data are in contrast with other national studies, where different pathways were observed in academies, such as Switzerland, where the RAE was not presented in national Under 17 and 19 [37] and in national level selection [29]. Overall, the present data underlined that the socio-cultural context (i.e., depth of competition, attraction level/sports popularity, and country-specific differences) might affect the RAE at the female youth level. On the contrary, no statistically significant differences in birth quartile distribution were observed in National Senior team players. This result contrasts a previous study in the same national context [27]. Nevertheless, the difference in sample selection (i.e., the first one considering the Italian national teams and the second one considering the Italian national teams) may partially explain these results. On the other hand, data corroborated previous results underlined as RAE disappears from youth to senior level [20, 24].

The analysis of the playing position provides additional information about the mechanisms of the RAE in female football. Data suggested asymmetries in relative age within defenders and midfielders Under 17 with medium effect size. In contrast, no RAE was observed in the Under 19 and national senior team. Focusing on the Q1 vs. Q4 comparison, data suggested that goalkeepers, defenders, and midfielders born in Q1 were about three times more likely to be selected in the Under 17. However, it is necessary to notice that the quartile distribution favoured the Q1 and Q2 in Under 19 for all playing positions, which is likely due to the small sample size affects the power of statistical analysis. The role of playing position has showed that RAE increases particularly among female goalkeepers and defenders in Spain [38] or the U.S. [22], defenders and in midfielders and forwards in Switzerland [37], and only in midfielders when considering Women’s Football World Cup rosters (i.e., Under 17 and 19) [20]. Even if the specific mechanisms explaining these trends have yet to be determined, the increased physical demands of those player positions [39] may have affected the observed RAE. Overall, it is possible to suggest, according to male literature [18, 40, 41], that these differences may be based on the country-specific differences and playing styles [23]. With this analysis of RAE both in youth and senior National teams, it is possible to suggest the small/medium effects observed in Under 17 and 19 age groups did not lead to ‘knock-on effects’ at senior level that have been previously shown, for instance, in the male national Italian context [14].

When analysing the birth quartile youth-to-senior transition, data detect a partial influence of the underdog hypothesis on this transition, underlining a reversal of RAE advantage at the senior level. Players born in Q4 showed a higher conversion rate than those born in Q1 (i.e., Q1 = 16.4% vs. Q4 = 25.0%). Of note, Q2 presented a similar trend to Q4. This result was in line with the Q2 ‘conundrum phenomenon’ [42] observed in different female sport context [17], where a spike in Q2 birth distribution was observed at the senior level. Supporting this idea, the quartile distribution birth in Q2 increased from the youth to the National Senior team (i.e., from 24.5% to 30.2%). The comparison between the U17 quartile distribution and youth-to-senior successful players provided additional data about the underdog hypothesis. Data suggested a significantly skewed compared with the U17 distribution. However, the only difference among quartiles was between Q4 and Q3. On the other hand, players in Q4 were approximately five times more likely to achieve a National Senior team than Q3 players. Again, the lower percentage of successful players may affect these results. Nevertheless, these results merit additional investigation in future research. Focusing on the comparison between Q1 and Q4, research on male football players [14, 43] detected a lower quartile transition rate compared to this data (i.e., Q1 = 7% vs. Q4 = 11.1%) in a male Italian sample. Nevertheless, while males had a higher likelihood of relatively younger players making the transition youth-to-senior successfully (about three and four times), our data did not show this difference [14, 33]. The lower turnover in female rosters and the lower sample size of this study may explain this difference. Moreover, the lower depth of competition and popularity of football among females in Italy may affect this result.

Within these analyses regarding quartile youth-to-senior transition, we partially supported the potential underdog benefits in female football. Indeed, the data did not provide a difference among quartile transition rates but only a tendency to favour relatively younger players, especially for Q4 but not for Q3 players. It is possible to suggest that relatively younger players overcame the initial birth date disadvantages [44] and had a greater chance of successfully transitioning from youth-to-senior career. Researchers emphasised that relatively younger players may develop superior performance [34] and psychological skills [45] during youth. On the other hand, relatively older players have higher injury rate [33, 44, 46] and develop pressure to be early-birth players that over time may be detrimental for overall well-being [34]. However, partially supported by our data, only a small percentage of relatively younger athlete benefits from overcoming the pitfalls of the interaction between birth and selection date [34].

Some limitations should be underlined when interpreting this study. We could only record birth dates and not anthropometric data, maturational status and performance variables useful for better describing both the youth-to-senior transition rate and RAE. Moreover, we considered all the players selected in the Under 17, Under 19 and national senior teams without considering the number of times that they were selected in the different age-groups. Finally, we considered friendly or official matches without distinction. These data may provide additional information about female soccer’s youth-to-senior transition rate and RAE.

## Conclusion

The present results add a broader overview of the youth-to-senior transition rate, the RAE, and the underdog hypothesis in female football literature. Overall, our data suggested that only a few players (17%) selected in the youth reached the national senior team, indicating a high turnover in Italian youth teams’ rosters, and the presence of the RAE in both Under 17 and 19 with a playing position’s effect in the younger category. Moreover, given the absence of an evidence of difference among quartiles in transition rates, our data partially supported the underdog hypothesis and it may be linked with a more substantiated knock-on effect in this female context.

This highlights the possible gender-specific mechanisms that occur, such as sports popularity, selection opportunities, and differences in the timing of maturation. Thus, as female football provision continues to advance, it is important to learn from the many pitfalls of male talent pathways and appreciate that women and girls require different organisational structures and settings. Talent selection and deselection process should take in account and favour player’s potential development rather than current performance, attenuating the temporary disadvantage of relative younger players.

However, to our knowledge, this is the first study in female football that analysed birthdate quartiles distributions related to youth-to-senior transition rates, and future studies are necessary to investigate more in deep this issue.

## Supporting information

S1 Fig Birth quartile percentage distributions presented individually for all playing positions, goalkeepers, defenders, midfielders and forwards. (TIF)

S1 Data (CSV)

S1 Text (TXT)
