# Supplementary figures and images for: Youth-to-senior transition in women’s and girls’ football: Towards a better understanding of relative age effects and gender-specific considerations
Source: PLoS One. 2023 May 4;18(5):e0283781. doi: 10.1371/journal.pone.0283781 (PMC10159103; doi:10.1371/journal.pone.0283781)

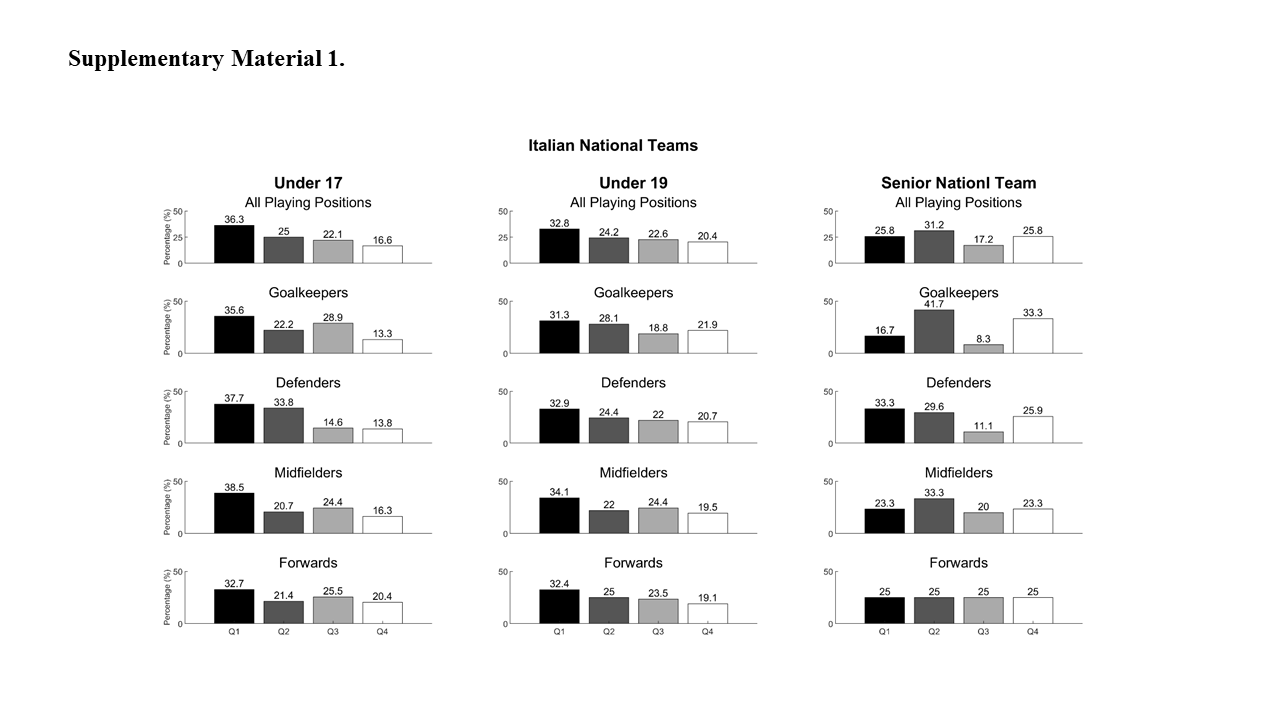

Supplement: S1 Fig — (TIF) [file pone.0283781.s001.tif]
